# The complement system in glioblastoma multiforme

**DOI:** 10.1186/s40478-018-0591-4

**Published:** 2018-09-12

**Authors:** T. A. M. Bouwens van der Vlis, J. M. Kros, D. A. M. Mustafa, R. T. A. van Wijck, L. Ackermans, P. M. van Hagen, P. J. van der Spek

**Affiliations:** 1 Department of Bioinformatics, Erasmus University Medical Center, Wytemaweg 80, 3015 GE Rotterdam, The Netherlands; 2 Department of Neurosurgery, Maastricht University Medical Center, P. Debyelaan 25, 6229 HX Maastricht, The Netherlands; 3 Department of Pathology, Erasmus University Medical Center, Wytemaweg 80, 3015 GE Rotterdam, The Netherlands; 4 Department of Internal Medicine, Erasmus University Medical Center, Wytemaweg 80, 3015 GE Rotterdam, The Netherlands; 5 Department of Immunology, Erasmus University Medical Center, Wytemaweg 80, 3015 GE Rotterdam, The Netherlands

## Abstract

The human complement system is represents the main effector arm of innate immunity and its ambivalent function in cancer has been subject of ongoing dispute. Glioma stem-like cells (GSC) residing in specific niches within glioblastomas (GBM) are capable of self-renewal and tumor proliferation. Recent data are indicative of the influence of the complement system on the maintenance of these cells. It appears that the role of the complement system in glial tumorigenesis, particularly its influence on GSC niches and GSC maintenance, is significant and warrants further exploration for therapeutic interventions.

## Introduction

**Fig. 1:**
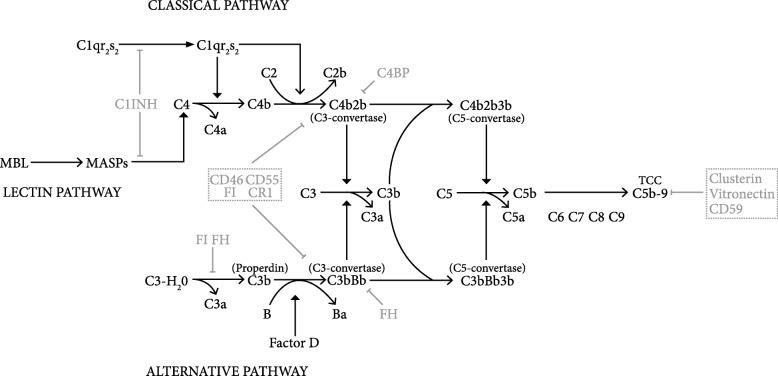
Schematic and simplified representation of the complement system. Complement regulatory proteins, both fluid-phase and membrane- bound are coloured light grey. *C1INH: C1-inhibitor; CD46: Membrane Cofactor Protein; CD55: Complement decay-accelerating factor; FI: Complement factor I; CR1: Complement receptor type 1; FH: Complement factor H; C4BP: C4bbinding protein*

Traditionally, the human complement system is regarded to be a main effector arm of the innate immunity and comprises around thirty soluble and membrane-associated proteins. Innate immunity forms the first line of defense against invading micro-organisms, and is ancient compared to the adaptive immune system. Analogues of components of the mammalian alternative complement pathway have been identified in deuterostomes/protostomes over 1000 million years ago, whereas the first molecules from the jawed-invertebrate specific adaptive immune system emerged at least 400 million years later [63]. After breaching the host’s environmental barriers, invading microbes are detected by the pattern recognition molecules (PRM) of the classical (C1q) and lectin (MBL, ficolins) complement pathways (CP, LP) [75, 96]. (Fig. 1). Further, activation of the complement cascade through the AP is achieved through insufficient inhibition of spontaneous hydrolysis of C3 (C3-H20) by the microbe (AP). All three activation pathways converge at the level of C3 which, after formation of the C3 and C5 convertase complexes, continues with the formation of the terminal complement complex (TCC) either as the pore-like membrane attack complex (MAC) or as cell-activating sC5b-9 [51]. MAC assembly in the cell membrane causes prompt colloid osmotic lysis [56, 59].

Over a century after the initial discovery of the complement system by Buchner et al. it was realized that the actions of complement are not constrained to an effector mechanism of the innate immunity, but are also involved in directing the adaptive immune response, angiogenesis, tissue regeneration, fat metabolism and development of the central nervous system (CNS) [75]. The complement system acts as intricate immune surveillance system that is able to discriminate between healthy host tissue, cellular debris, apoptotic cells and foreign intruders (Fig. 1) and contributes to a large variety of inflammatory-, immune-, ischemic-, age-related pathologic processes of the CNS [75]. A wide repertoire of specific complement inhibitors has been developed against a variety of diseases of which eculizumab, an antibody against C5 and C1 (C1-INH) became FDA approved for the treatment of paroxysmal nocturnal hemoglobinuria, atypical hemolytic uremic syndrome and hereditary angiogoedema [1]. In recent years it became clear that many factors of the complement system are expressed in the brain [60, 93]. The production of the complement proteins in CNS is constrained to microglia, oligodendrocytes, astrocytes and, to a lesser extent, ependymal cells [93]. The complement system appears to be a key player in CNS homeostasis as complement effector mechanisms have been identified in neurogenesis and regulating synaptic pruning [54]. Various pathological conditions cause an imbalance between complement activation and inhibition.

Activation of the complement system contributes to a wide variety of CNS diseases including Alzheimer disease, CNS inflammation, traumatic brain injury and tumors [6, 55]. The cross-link between inflammation and cancer is generally accepted as chronic and insidious inflammation is recognized to play decisive roles at different stages of tumor development, including initiation, promotion, malignant conversion, invasion, and metastasis [28, 52]. The actions of the immune system against tumors in progress can be referred to as the cancer immunoediting process, which is composed of three distinct phases: elimination, equilibrium and escape. During the elimination phase and the equilibrium phase the immunological response is able to prevent tumor progression. In contrast, during the escape phase acquired adaptations of malignant cells and the host immune system response allow for expansion of the tumor cell population [94]. By acting as an intrinsic effector mechanism and by forming a functional bridge between the innate and the adaptive immune system, the complement system is an integral component of the antitumor immune response [94]. Complement activation following recognition of damage-associated molecular patterns (DAMPs) expressed by tumor cells, or improper regulation, allow for a potent anti-tumor response [37]. The potent antitumor response by complement has been utilized for antibody-based cancer immunotherapies by eliciting complement-dependent cytotoxicity, exemplified by the use of rituximab and ofatumumab in the treatment of B cell lymphomas and chronic lympocytic leukaemia, respectively [89]. However, the complement system also shows another face. Recent pre-clinical cancer models showed that the activated complement system contributes to a tumor facilitating micro-environment [1]. This adverse capacity seems to be a consequence of imbalanced, rather than physiological, complement activation [74]. Various studies have reported significant reductions of orthotopic tumor growth following complement system inhibition within the cascade [74]. Further, diverse complement effectors are implicated in other cancer-related phenomena as sustained proliferative signaling, angiogenesis and invasion and metastasis [33, 74].

Contributory to treatment resistance of glial neoplasms is the presence of glioma stem-like cells (GSCs) [85]. GSCs reside in specific anatomical niches within the tumor and propagate glioma repopulation by converting into either a differentiated tumor cell, or a new cancer stem cell [46]. The maintenance of GSCs requires specific intrinsic factors within the cells and various paracrine cues from adjacent cells [46]. The complement system represents an as yet unidentified effector in GSC maintenance, and unraveling its interplay will reveal new targets for therapeutic intervention.

## Complement and GSC maintenance: Intrinsic regulation

**Fig. 2:**
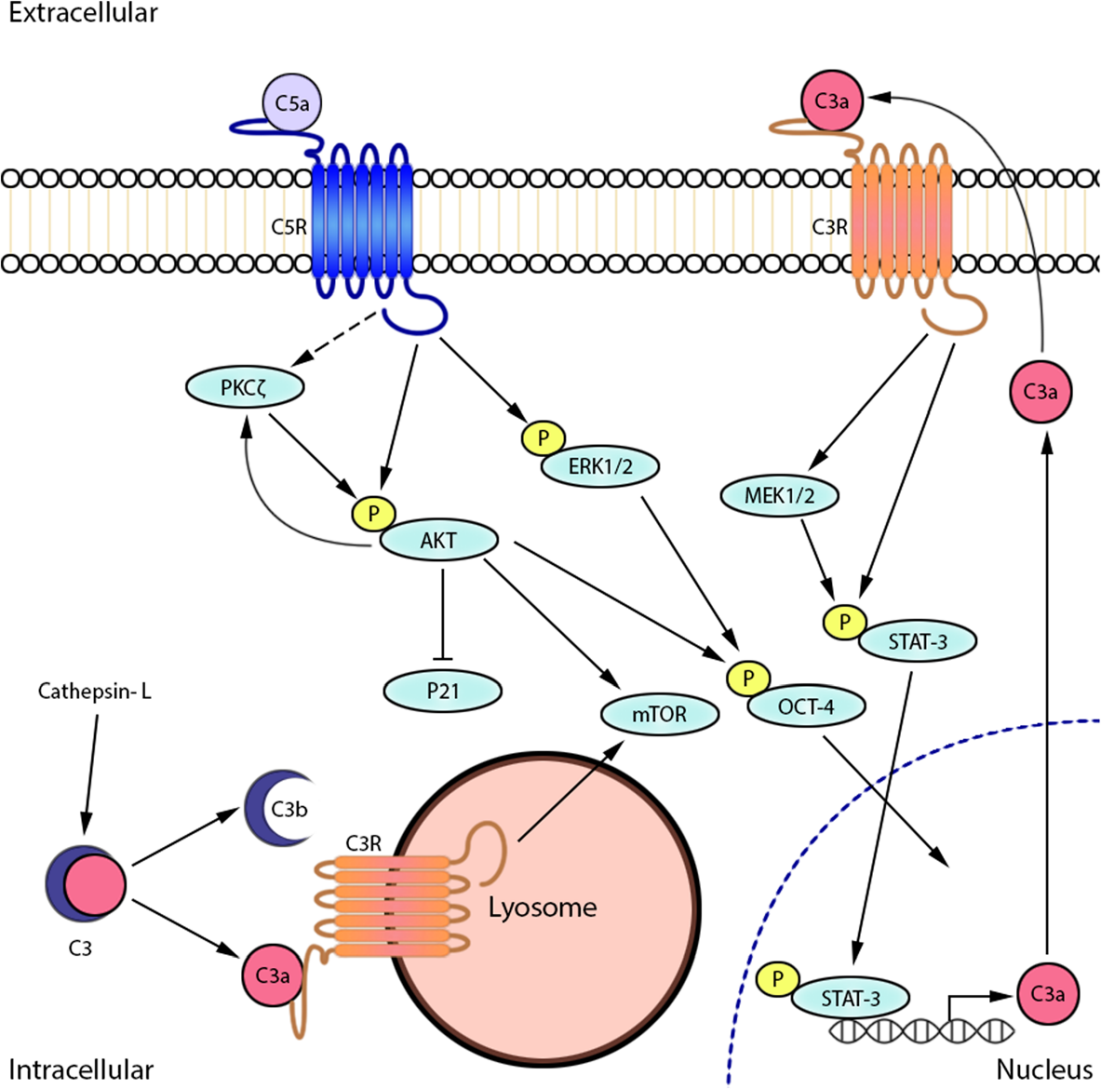
Proposed interaction of complement C3a and C5a with GSC regulatory mechanisms. C5a-C5aR interaction activates PI3K/Akt/mTOR signaling and PKCζ but suppresses p21 with subsequent OCT-4 activation. Intracellular activation of C3a by cathepsin-L may occur, thereby sustaining basal mTOR activation. Either intracellular or extracellular derived C3a phosphorylates STAT-3 and causes an increase of SOX-2 expression

Factors that are involved in GSC maintenance comprise of metabolic, genetic and epigenetic regulatory mechanisms [90]. Although the mechanisms underlying GSC plasticity are largely unknown, several intrinsic regulatory mechanisms are known to be involved in reprogramming differentiated GBM cells into stem-like cells. Among these are Sex Determining Region Y -Box 2 (SOX-2) [88], signal transducer and activator of transcription 3 (STAT-3), octamer-binding transcription factor 4 (OCT-4) and mammalian target of rapamycin (mTOR) signaling [23, 82]. The GSCs maintain their multipotent state through autocrine stimulation of the C3a- and C5a-receptors on the plasma membrane by secretion of alternative pathway C3-convertase components (C3, factor D and factor B) and subsequent extracellular cleavage of C3, as observed in resting T-cells (Fig. 2) [87]. The C3 and C5 convertases (Fig. 1) are responsible for the release of their respective bioactive fragments C3a and C3b, and C5a and C5b. The anaphylotoxins C3a and C5a signal through the G protein coupled receptors C3aR and C5aR (CD88) respectively. Interaction of several downstream signal transduction pathways followed by C3aR and C5aR activation with recognized GSC regulatory mechanisms effectors may therefore aid to GSC maintenance. Figure 2 presents a schematic overview of the interaction of autocrine derived complement with GSC regulatory mechanisms. C3a-C3aR interaction activates STAT-3 and causes an increase of Wnt2b and SOX-2 expression in a serine protease MAPK dependent fashion, as was shown in a model of retinal regeneration [36]. C5aR1 activation contributes to the maintenance of pluripotency of OCT-4 positive human induced pluripotent stem cells (hPSC) through extracellular signal-regulated protein kinases 1 and 2 (ERK1/2) activation [35]. In vitro administration of recombinant C5a to C5aR expressing gastric cancer cells promotes the activation of PI3K/Akt and downregulates p21 activation [11]. Inhibition of p21 is a key mechanism of GSC self-renewal and prevention of differentiation [99].

Further, complement C3 activation is not limited to the extracellular space because intracellular C3 activation is an ubiquitous event within human cells [50]. Resting T-lymphocytes contain intracellular pools of C3 that are activated by cysteine protease cathepsin-L. This ‘tonic’ intracellular C3a generation engages C3aR present on lysosomes and sustains the basal mammalian target of rapamycin (mTOR) activation required for T-cell survival [50]. Activation of the PI3K/Akt/mTOR signaling cascade is involved in GSC phenotype maintenance [18]. In addition, GBM tissue shows the profound presence of cathepsin L, which has been shown to negatively affect apoptosis and promotes invasion [104]. Intracellular C3a activation by cathepsin-L may provide for an as yet unidentified additional intrinsic GSC effector mechanism. Noteworthy is the critical role of autocrine derived C5a-C5aR1 signalling in affecting the fate of Neural Progenitor Cells (NPCs). C5a was found actively secreted within neural rosettes [16]. Atypical protein kinase C zeta (PKCζ) dependent C5a-C5aR1 signaling regulates the apicobasal polarity of the NPC, thereby maintaining symmetrical self-renewing cell division [16]. PKCζ inhibition reverses C5a-C5aR induced p42/44 phosphorylation (ERK) and attenuates the mitotic activity of NPCs [16]. PKCζ is overexpressed in GBM cells and its downregulation inhibits in vitro migration and invasion of GBM cells. Another mechanism through which the GSC could generate C5 in order to self-activate C5aR is by expressing cell membrane-bound serine protease, as was found in different cancer cell types [62].

## Complement and GSC maintenance

The neuropil constitutes the extracellular matrix of the brain in which endothelial and perivascular cells, microglia, tumor-associated macrophages and non-neoplastic astrocytes are present and networks of cytokines and growth factors are active [32]. Together with neoplastic cells the microenvironment coalesces into the tumor mass. GSC are enriched in areas of hypoxia, in perivascular niches and at the invasive edge of the tumor [46]. A graphical representation of the various cells involved is provided in Fig. 2. The actions of the complement factors within the various glioma niches are summarized in Table 1.

**Table 1:** Complement protein in the GBM tumor

Complement proteins	Niche	Mechanism	Proposed effect
C1q	GSC	Wnt activation	Stimulate GSC differentiation
	Perivascular	Priming SDF-1 gradient	GSC migration
	Perivascular	gC1qR interaction	Potentiate tumor cell invasiveness, chemoattractant
	Perivascular	cC1qR interaction (IL-8, MCP-1 and IL-6 secretion)	GSC migration
	Microenvironment	GAM-M2 induction	Immunosuppression
	Invasive	gC1qR (Bradykinin induction)	Tumor cell invasiveness
MBL	Microenvironment	GAM-M2 induction	Immunosuppression
C3	Microenvironment	MDSC recruitment	Immunosuppression
C3b	Microenvironment	Treg induction (ligand for CD46)	Immunosuppression
	Microenvironment	GAM-M2 induction	Immunosuppression
C3a/C3aR	Hypoxic	STAT-3 activation	GSC maintenance
	GSC	mTOR activation	GSC maintenance
	Hypoxic	NOX-4 activation, (STAT-3, HIF-α)	GSC maintenance
	GSC	SOX-2 activation	GSC maintenance
	Microenvironment	Chemotaxis immune cells	
CD46	Hypoxic	Jagged-1-Notch disruption	GSC maintenance
	Perivascular		
C5a/C5aR	Invasive nice	PKCζ activation	Potentiate tumor cell invasiveness
	GSC	PI3K/Akt/mTOR	GSC maintenance
	GSC	P21 inhibition	GSC maintenance
	GSC	OCT-4 expression	GSC maintenance
	Perivascular	MMP-9, MT1-MMP activation	Increase tumor cell invasiveness
	Perivascular	MCP-1 secretion	Increase tumor cell invasiveness
	Perivascular	TGF-β	GSC differentation into vascular pericytes
	Perivascular	NO secretion (iNOS/eNOS induction)	GSC maintenance
	Perivascular	VEGF expression	Vascular tube formation
	Microenvironment	Chemotaxis immune cells	
	Microenvironment	Treg induction (High concentration)	Immunosuppression
	Microenvironment	GAM-M1 activation (balanced C activation)	Anti-tumor response
	Microenvironment	MDSC recruitment (ROS)	Immunosuppression
C5b-C9	Perivascular	bFGF release	GSC dedifferentiation

### Hypoxic niche

The inadequate neo-angiogenesis in GBM results in hypoxic areas within the tumor. The neoplastic cells respond to low oxygen levels by the expression of members of the hypoxia inducible factor (HIF) family of transcriptional factors [49]. HIFs are upregulated in GSC and its forced expression induces a stem-cell like phenotype in glioma cells [49]. Transcriptional targets of HIFs include angiogenic genes like Vascular Endothelial Growth Factor (VEGF) as well as stem cell markers [49]. Areas of hypoxia optimally accommodate complement activation as provide for damage-associated molecular patterns (DAMPs) that are recognized by C1q. Hypoxic conditions induce (HIF-dependent) down-regulation of complement regulatory genes CD55, CD46 and factor H and upregulate C3, C3a and C3aR and enhance C3a-C3aR engagement [27, 66]. The constituents of the complement system have been identified to interact with HIF associated signaling pathways and may therefore act as an additional effector mechanism in HIF dependent GSC survival, self-renewal and tumor growth. Firstly, the complement system contributes to facilitate HIF transcription through STAT-3 activation that is critical for the transcription of HIF-1α in GSCs and tumor-associated myeloid cells [69]. The production of reactive oxygen species, as a result of overexpression of nicotinamide adenine dinucleotide phosphate oxidase 4 (NOX-4), was identified as the molecular mechanism underlying hypoxia-induced STAT-3 activation in GBM cells [103]. In a model of renal ischemia/reperfusion injury, oxidative stress induces an increased expression of NOX-4 in tubular cells and NOX-2 infiltrating monocytes and myeloid dendritic cells [84]. This effect is dramatically reduced after the administration of the complement 1-inhibitor (C1-INH). In vitro administration of C3a to cultured proximal tubular cells induces NOX-4 expression regardless of hypoxic conditions [84].

Secondly, through C3aR and C5aR interaction on the GSC, complement may provide for additional signal transduction pathways for PI3K- or mitogen-activated protein kinase (MAPK)/ERK1/2-dependent HIF-1α protein translation [68, 69]. HIF-1α and the components of the complement cascade converge at the level of the Notch signaling pathway. Notch activation restricts glioma cell differentiation and stimulates astrocytes into a neural stem-like cell state [69]. HIF-1/2α driven GSC maintenance requires Notch signaling [69]. In resting T-cells, CD46 sequesters the Notch ligand Jagged-1, thereby preventing the interaction between Jagged-1 and Notch that activates T-cells [48]. Hypoxia-mediated downregulation of the expression of CD46 or CD46-C3b interaction following complement activation may allow for Notch-Jagged-1 interaction. A direct contribution of CD46 downregulation in maintaining the undifferentiated state of the GSC remains to be elucidated. C3a inhibits SDF-1α induced neuronal differentiation of NPCs through ERK1/2 phosphorylation regulation [83]. SDF-1α is a HIF-1α target gene in GBM cells [22]. Importantly, SDF-1α induces recruitment of bone-marrow derived CD45+ myeloid cells, endothelial and pericyte progenitor cells to GBM [22].

Lastly, HIF-1α modulated, Wnt/ β-catenin activation has been identified to stimulate GSC differentiation and therefore promotes a less-aggressive, neuronal tumor phenotype. Subsequent β-catenin mediated Notch inhibition further allows for GSC differentiation [71]. The role of Wnt activation in regulating the GSC state remains controversial as many reports claim that Wnt activation promotes GSC maintenance and expansion [42]. C1q is an activator of canonical Wnt signalling through binding with the Fz-receptor and subsequent induction of C1s dependent cleavage of low-density lipoprotein receptor-related protein 6 (LRP6) [61]. Intriguingly, C1q-induced activation of Wnt signalling attenuates the proliferation of muscle stem cells [61].

### Perivascular niche

As a result of vigorous and abnormal angiogenesis, local regions of hypoxia develop with subsequent complement activation, facilitating complement mediated GSC regulation. The actions of the complement system extend into the perivascular niche, where GSC survival and angiogenesis are mediated. In the adult mammalian brain, neuronal stem cells reside in two germinal niches of which the subventricular zone is highly vascularized. Adult stem cells lie in close proximity to the vasculature, where communication occurs through direct cell-cell interactions and soluble secreted cues [81]. In contrast to germinal niches, where the rate of stem cell proliferation is low, the perivascular niche within GBM contributes to the generation of the GSCs and tumor growth [9]. Besides endothelial cells (EC) and GSCs, major cell types that have been recognized to reside in the perivascular niche include pericytes, immune cells, fibroblasts and astrocytes, all of which provide an additional biological source of complement proteins [9, 93]. The complement system establishes an active interplay with the perivascular niche constituents and GSCs and fulfills an active role in the migration of GSC and promotes angiogenesis.

Several components of the (activated) complement system are powerful chemoattractants attracking blood borne cells and GSCs to the perivascular niche [75]. The complement system potentiates the migration of GSC towards the perivascular niche just like it does to mesenchymal stem cells (MSCs), NPCs and hematopoietic stem/progenitor cells (hPSC). In GBM, GSCs move towards the tumor vasculature and engage in cell-cell contact [9]. The interaction of Stromal Cell-Derived Factor 1 (SDF1) with its receptor C-X-C Motif Chemokine Receptor 4 (CXCR4) is operative to guide the tumor cells to the peri-endothelial space [72]. C1q primes chemotactic SDF-1-dependent migration of human umbilical cord blood derived- Mesenchymal Stem Cells (MSC), in part by upregulating the expression of CXCR4 and through interaction with its globular heads binding receptor (gC1qR), which is ubiquitously expressed [70]. The activated complement system potentiates the SDF1-CXCR4 chemotaxis, independent of C3aR, as has been observed in hematopoietic stem/progenitor cells (HSPCs) [38]. C3a modulates concentration-dependent SDF1-CXCR4 induced migration of NPCs [83]. C5a attracts MSCs in a C5aR dependent fashion and its degradation product C5a_desArg_ causes an increased secretion of MMP-9 and MT1-MMP, creating a highly proteolytic microenvironment in favor of cell-migration [79]. In addition, IL-8 secreted by ECs stimulates GSC migration and maintains its stemness properties, in part by upregulating the expression of its cognate receptors CXCR1 and CXCR2 [39]. The interaction of C1q with its receptor present on the EC (cC1qR) initiates the release of IL-8, macrophage chemoattractant protein-1 (MCP-1) and IL-6, that contribute to the homing of GSCs and MSCs [92, 97]. IL-8 mediated GSC homing and maintenance is amplified by C5a as observed in whole blood cells and in human dermal microvascular endothelial cells (HVEC-d) [97]. Lastly, C5a contributes to GSC migration by increasing the expression of MCP-1/CCL2, as observed in HVEC-d in a dose dependent matter [102].

### Complement modulated Perivascular niche-GSC interaction

GSCs form intimate contacts along the length of the endothelial tube, essential for their survival and inducing secretion of soluble factors by ECs that keep the GSCs in an undifferentiated state [80]. Several pathways constitute EC orchestrated GSC regulation, including the transforming growth factor-β (TGF-β) pathway [80]. EC-derived TGF-β induces the differentiation of GSC into pericytes that contribute to vascular formation in GBM [12]. Crosstalk of TGF-β signaling and complement activation is observed in various cells. C5a has been shown to upregulate TGF-β transcript expression, and vice-versa TGF-β upregulates the expression of C5aR [29]. Further, TGF-β and C5a signaling converge downstream at the level of SMAD independent pathways including, PI3K/AKT/mTOR and MAPK/ERK1/2 signaling pathways [77, 105]. A second pathway through which the GSC phenotype is maintained is the nitric oxide (NO) signaling [10]. The biological source of NO is EC-derived by the expression of endothelial NO synthase (eNOS) or alternatively, through the expression of inducible NOS (iNOS) by the GSC [10]. Activation of eNOS requires phosphorylation of AKT, suggestive of a contributory role for the activated complement system [21]. The complement system mediates the expression of iNOS and NO levels as shown in models of gastrointestinal ischemia by inhibition of C3 and C5 [57]. Lastly, EC derived basic Fibroblast Growth Factor (bFGF) induces the reversion of differentiated GBM cells [25]. However, the underlying mechanism resulting in functional expression of bFGF remains poorly defined, given that hypoxia does not induce bFGF expression in human vascular smooth muscles cells [7]. Interestingly, small amounts of C5b-9 (membrane attack complex) releases bFGF from human umbilical vein endothelial cells (HVEC) [4]. Active and inactive forms of C1s are found to form aggregates with bFGF, thereby reducing its activity [76].

### The complement system influences GSC mediated angiogenesis

Indications for the close interaction of GSC and endothelial cells emerged from the finding that xenotransplanted tumors derived from GSCs were characterized by widespread angiogenesis that was not encountered in their non-GSC counterparts [3]. GSCs secrete VEGF and treatment with bevacizumab blocks the pro-angiogenic effects of VEGF by hampering microvascular endothelial cell migration and vascular tube formation and inhibiting the growth and vascularity of GSC derived xenotransplants [3]. Bioactive complement products have been identified as important effectors in pathological neovascularization in age-related macular degeneration (ARMD), diabetic retinopathy, and retinopathy of prematurity [100]. Interaction of C5a with its receptor C5aR1 induces VEGF expression in a dose-dependent matter in retinal pigmented epithelium (RPE) in-vivo and in-vitro [15]. The induction of oxidative stress in RPE cells reduces the surface expression of DAF, CD55 and CD59 and impairs complement regulation at the level of factor H, resulting in complement activation and complement-dependent VEGF expression [91]. Consequently, inhibiting the AP using a recombinant factor H reduces the expression of VEGF and subsequent angiogenesis in a mouse model of choroidal neovascularization [91]. Conversely, the inhibition of VEGF causes a decrease of the complement inhibitory proteins (CIPs) factor H, CD46 and CD59 in human RPE-cells and glomerular endothelial cells through VEGFR2/PKC-α/CREB signaling [44]. These observations imply that VEGF protects neo-angiogenesis by local inhibition of the complement system. It remains undetermined whether complement activation directly contributes to VEGF expression or VEGF suppresses complement activation through CIP induction. In a mouse model of ovarian cancer, C3 and C5aR were shown to be closely involved in neo-angiogenesis [63]. Tumors derived from partial C3, C5aR and complete C5aR knock out mice displayed decreased microvascular density compared to their WT-littermates [63]. Additional in vivo assays showed significant impairment of angiogenesis for complete C3 and C5aR knock-out mice. Interestingly, direct functional effect of C5a comparable to VEGF-A on tube formation of endothelial cells was also observed. This effect was found to be reversible using the C5aR inhibitor PMX-53. PMX-53 also significantly impaired VEGF_165_ mediated HMEC tube formation [63]. In addition to C3 and C5aR, microvascular density was significant decreased in tumors in C1q deficient mice bearing a syngeneic B16 melanoma compared to their WT-littermates [8].

## Complement and immune cell crosstalk in the perivascular niche

Activation of the complement system by means of C3a and C5a plays an important role in the inflammatory process by recruiting immune cells such as mast cells, monocytes, macrophages, neutrophils, MDSCs and adaptive immune cells including T cells [75]. The BBB consists of highly specialized endothelial cells that communicate with pericytes and astrocytes to protect the CNS from the chemical variations in the bloodstream, and establishes a strictly controlled interface for immune cell trafficking. In GBM the BBB’s integrity is disrupted due to the abnormal tumor microvasculature, resulting in an increased vascular permeability and consequently, an increase in immune cell infiltration including monocyte-derived cells, microglia and T-lymphocytes [19, 24]. C5a/C5aR neutralization alleviates the BBB breakdown in models of traumatic brain injury and systemic lupus erythematodus and it is likely that the activated complement system also affects the BBB in GBM, with possible consequences for the passage of immune cells [40].

### Lymphocytic infiltration and PD-1

In glioma, tumor infiltrating lymphocytes (TILs) consisting of CD4+ and CD8+ cells are present [65]. Glioma TILs show a predominant regulatory T-cell population (CD4 + CD25 + Foxp3+) [65]. Regulatory T cells (Tregs) are believed to be the primary regulators of immunosuppression in the glioma microenvironment [65]. The proportions of CD3+ and CD8+ over Foxp3+ cells reportedly correlate with the clinical course of GBM patients [78]. The activated complement system by means of CD46 may account for an increased proportion of Tregs. The C3 cleavage fragment, C3b, is a natural ligand for CD46 on T cells. Stimulation of naïve CD4+ T cells with anti-CD46 monocolonal antibodies (mAb) or C3b dimers in the presence of IL-2 induces a differentiation towards a IL-10 producing type 1 regulatory T cell (Tr1) [45]. However, CD4+ Foxp3+ regulatory T cells present in GBM are predominantly thymus derived (tTregs) rather than peripheral induced IL-10 producing regulatory T-cells [95]. In the presence of CD46 stimulation, cell contact-mediated tTreg function is impaired [47]. Instead, tTregs differentiate to IL-10 secreting Tr1 cells [47]. In several human cancers a potent immunosuppressive subpopulation of IL-10 producing Tregs has been identified and these Tregs suppress CD8+ T-cell effector functions which is associated with poor survival [64].

In models of melanoma and non-small cell lung cancer combined with genetic ablation or mAb blocking of programmed death 1/programmed death ligand 1 (PD-1/PD-L1) and C3aR appears to be more effective in restraining tumor growth than only blocking PD-1 therapy alone [2]. In glioma, the expression of PD-L1 is correlated with glioma grade and has been identified as a negative prognostic factor. Recently, therapeutic blockade of PD-1 in the GL-261 murine glioma model induced an impressive prolonged survival, with TILs showing a shift towards CD8+ T cells [20]. The dual role of complement activation in the tumor micro-environment was illustrated by tumor progression in tumor-bearing mice with either high- or low C5a-producing syngeneic lymphoma cells [30]. High C5a producing tumors showed a significant increased tumor progression associated with an overall decrease CD4+ and CD8+ T cells in the tumor [30]. Further, it was shown that in vitro polarization of CD4+ cells is observed to be C5a concentration dependent. A low C5a concentration promotes Th1 cell differentiation while high concentrations (> 500 ng/ml) promotes Treg induction [30]. Taken together, imbalanced complement activation may be associated with an immunosuppressive micro-environment and is therefore contributory to tumor progression.

### Glioma associated microglia and macrophages (GAMs)

Glioma associated microglia and macrophages (GAMs) are considered to be the most prominent glioma-infiltrating immune cells, constituting up to 30% of all immune cells within the tumor microenvironment. GAMs are recruited into the tumor microenvironment through various glioma derived factors that contribute to polarization from a tumor-suppressive to a tumor-promoting phenotype. Activated complement factors may contribute to the constant recruitment of GAMs to the tumor micro-environment, either directly, or through interactions with glioma factors, and influence their polarization. Several factors have been recognized to attract GAMs to the tumor environment, among which are MCP-1 and SDF-1 [31]. C1q and C5a have been shown to potentiate the actions of these chemokines [67, 70]. Upon lipopolysaccharide (LPS), IFNγ, or C3a and C5a induced activation (M1) GAMs produce inflammatory mediators, phagocytose tumor cells, present antigens to immune cells and induce a T-cell response [34]. In contrast, GAMs activated through factors such as CSF-1, IL-10, TGF-β and the complement opsonins C1q and C3b acquire a pro-tumorigenic phenotype (M2) which include promoting and facilitating immunosuppression, glioma proliferation and invasiveness [98]. Unbalanced complement activation induces Il-10 and TGF-β expression, thereby promoting a pro-tumorigenic phenotype [29, 47]. Within the scope of autoimmunity the complement opsonins C1q and MBL regulate macrophage polarization towards a M2 macrophage phenotype [5, 26].

### Myeloid-derived suppressor cells (MDSC)

Myeloid-derived suppressor cells (MDSCs) are a heterogeneous population of cells containing myeloid cells in various differentiation stages. MDSCs are found to be significantly increased in the peripheral blood of glioblastoma patients [73]. MDSCs are believed to elicit distinct immunosuppressive actions within the glioma micro-environment including generation of oxidative stress through the production of ROS and thereby inducing T-cell inhibition [24]. Intriguingly, in a TC-1 syngeneic model of cervical cancer in mice, pharmacological inhibition of C5aR resulted in an increase in CD8+ T-cell infiltration along with deceleration of tumor growth comparable to the effects of paclitaxel [53]. C5a mediated suppression of the antitumor CD8+ T-cell response is associated with an increase of MDSC in the tumor microenvironment and a subsequent increase in ROS production [53]. In a syngeneic mouse model for lung cancer, MDSCs appeared to be reduced in a subpopulation analysis of splenocytes after C5aR blockade [14]. Concordantly, in a murine model of ovarian cancer C3 silencing increases the number infiltrating CD8+ T-cells infiltrating the tumor by 10-fold and reduced the number of MDSCs by 80% [13]. However, the observed reduction in tumor growth was found to be independent on the number of CD8+ T-cells [13].

## Invasive niche

High-grade gliomas show aggressive invasiveness in two compartments: the perivascular space and the brain parenchyma [17]. The bradykinin and the SDF-1/CXCR4 axis act as a chemoattractant that guide glioma cells towards blood vessels [58]. The bradykinin-forming cascade and the classical complement pathway share many elements, including cross-activation, shared binding proteins and control mechanisms [43]. C1 inhibitor (C1 INH) inhibits the bradykinin-forming cascade at several levels: its local absence at the glioma invasive edge may further initiate the classic complement cascade by activating C1r [43]. Vice-versa, the presence of the ubiquitously expressed receptor for the globular head of C1q (gC1qR) allows for bradykinin production on the endothelial cell surface [43]. The activated complement system further stimulates glioma cell migration through facilitating local degradation of extracellular matrix proteins. In C5aR expressing colon and bile duc cancer cells C5a enhances cell invasiveness by increasing the expression of several matrix metalloproteinases (MMPs), including MMP-1 and MMP-9 in C5aR expressing colon and bile duct cancer cells [62]. Soluble C5b-9, which is as a pro-inflammatory mediator, induces MMP-2 expression by microglia upon activation [101]. The molecular regulation of MMP-2 and MMP-9 is poorly characterized. The activated complement system signaling may provide an alternative mechanism for the NF-κB mediated overexpression of MMP-2 and MMP-9 in glioma cells by means of C5a-C5aR [86]. C1q may activate the canonical Wnt/β-catenin signaling pathway, which increases MMP-2 and MMP-9 expression by glioma cells [41].

## Conclusion remarks and future directions

**Fig. 3:**
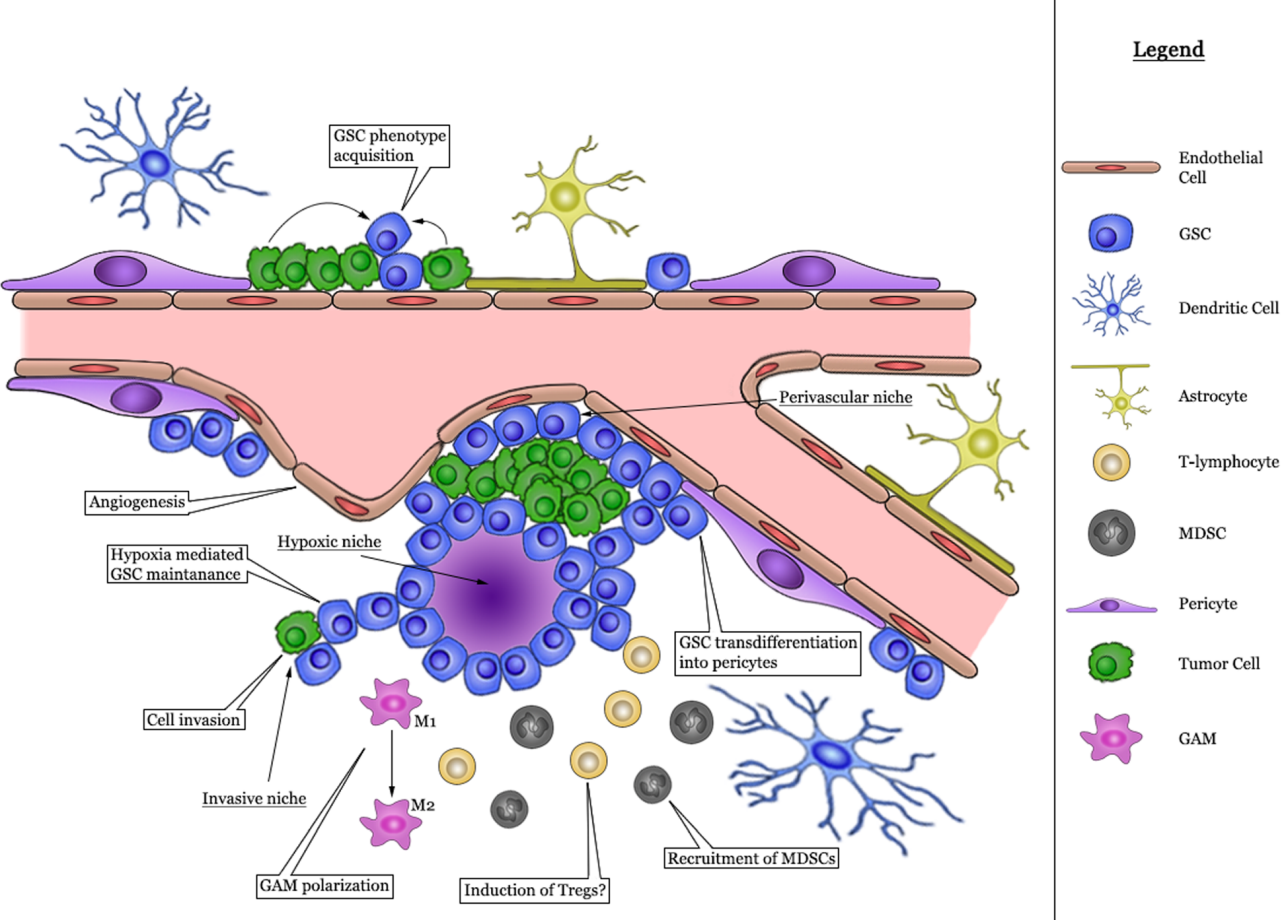
Graphical summary of the potential actions of the complement system in the glial tumor microenvironment

A wide variety of molecular pathways that are characteristic of the aggressive nature of GBM are known to be induced or modulated by the activated complement system (Table 1 and Fig. 3). The unbalanced activation in the tumor microenvironment may suppress the antitumor inflammatory response. There is increasing data that the complement system could serve as an unidentified intrinsic and extrinsic regulatory mechanism for GSC maintenance, thereby supporting treatment resistance. The activated complement system may contribute to evade the immune response, activate invasion and induce angiogenesis. Because several tumor promoting pathways are activated by complement the elucidation of the role of complement activation is necessary to discover new targets for therapy in malignant glioma.

## Abbreviations

AP: Alternative Pathway
BBB: Blood Brain Barrier
bFGF: basic Fibroblast Growth Factor
C1-INH: C1-inhibitor
C3aR: C3a Receptor
C5aR1: C5a Receptor-1
C5b-9: Membrane Attack Complex
cC1qR: collagen C1q Receptor (Calreticulin)
CNS: Central Nervous System
CREB: Cellular Transcription Factor
CSF 1: Colony Stimulating Factor 1
CXCR1: C-X-C Motif Chemokine Receptor 1
CXCR2: C-X-C Motif Chemokine Receptor 2
CXCR4: C-X-C Motif Chemokine Receptor 4
DAMP: Damage-Associated Molecular Pattern
EC: Endothelial Cells
eNOS: endothelial Nitric Oxide synthase
ERK1/2: Extracellular-signal regulated kinases
Fz: Frizzled
GBM: Glioblastoma Multiforme
gC1qR: Globular C1q Receptor
GSC: Glioma Stem-like Cell
HIF: Hypoxia Inducible Factor
HMEC: Human Mammary Epithelial Cells
hPSC: human induced Pluripotent Stem Cells
HVEC: Human Umbilical Vein Endothelial Cells
HVEC-d: Human Dermal Microvascular Endothelial Cells
IFNγ: Interferon-γ
LPS: lipopolysaccharide
LRP6: Low-density lipoprotein receptor-related protein 6
MAPK: Mitogen-Activated Protein Kinase
MBL: Mannose Binding Lectin
MCP-1: Macrophage Chemoattractant Protein-1
MMP-9: Matrix metallopeptidase 9
MSC: mesenchymal stem cells
MSDC: Myeloid-Derived Suppressor Cell
MT1-MMP: Membrane-Type 1 Matrix MetalloProteinase
mTOR: mammalian target of rapamycin
NO: Nitric Oxide
NOX-2: Nicotinamide adenine dinucleotide phosphate OXidase 2
NOX-4: Nicotinamide adenine dinucleotide phosphate OXidase 4
NPC: Neural Progenitor Cell
OCT-4: Octamer-Binding Transcription Factor-4
PD-1: Programmed Cell Death protein 1
PD-L1: Programmed Cell Death ligand 1
PI3K: Phosphatidylinositol 3-kinase
PKC-α: Protein kinase C- α
PKCζ: Protein Kinase C zeta
ROS: Reactive Oxygen Species
SDF-1α: Stromal Cell-Derived Factor 1α
SOX-2: Sex Determining Region Y -Box 2
STAT-3: Signal Transducer and Activator of Transcription-3
TGF-β: Transforming Growth Factor-β
TIL: Tumor Infiltrating Lymphocytes
VEGF: Vascular Endothelial Growth Factor
VEGFR2: Vascular Endothelial Growth Factor Receptor 2
Wnt: Wingless-related integration site
